# Supplementary figures and images for: Identification of olfactory genes of a forensically important blow fly, Aldrichina grahami (Diptera: Calliphoridae)
Source: PeerJ. 2020 Aug 5;8:e9581. doi: 10.7717/peerj.9581 (PMC7414772; doi:10.7717/peerj.9581)

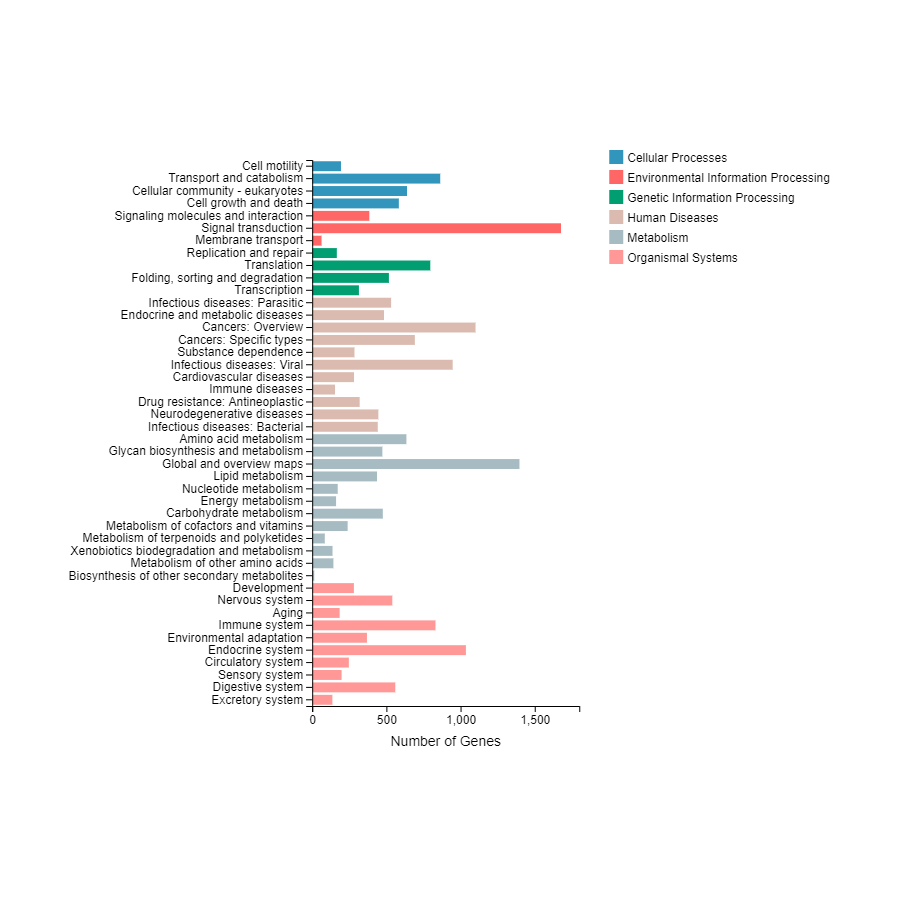

Supplement: Supplemental Information 1 — Unigenes are classifified into six categories: Celluar process, environmental information processing, Genetic information processing , Human diseases, and molecular function. KEGG pathway are shown on the right y-axis. The x-axis shows the number of genes that have a particular KEGG pathway. [file peerj-08-9581-s001.png]

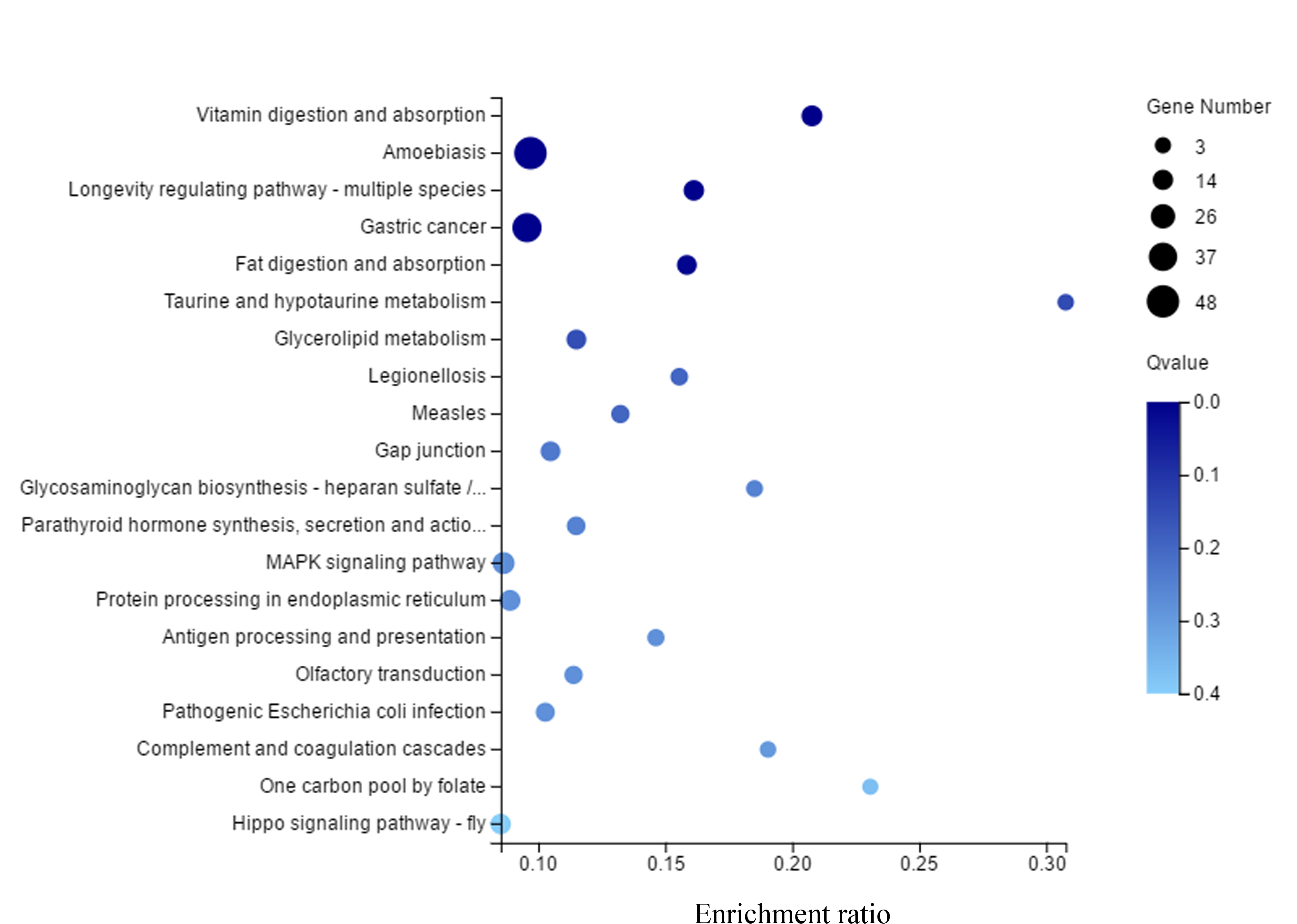

Supplement: Supplemental Information 2 — The x axis is the enrichment ratio (the ratio of the number of genes annotated to a certain target in the selected gene set to the total number of genes annotated to the item in this species, the calculation formula is enrichment ratio = termcandidate gene num/termgene num). The y axis is KEGG Pathway, and the size of the bubble indicates the number of genes annotated to a certain KEGG Pathway. [file peerj-08-9581-s002.png]

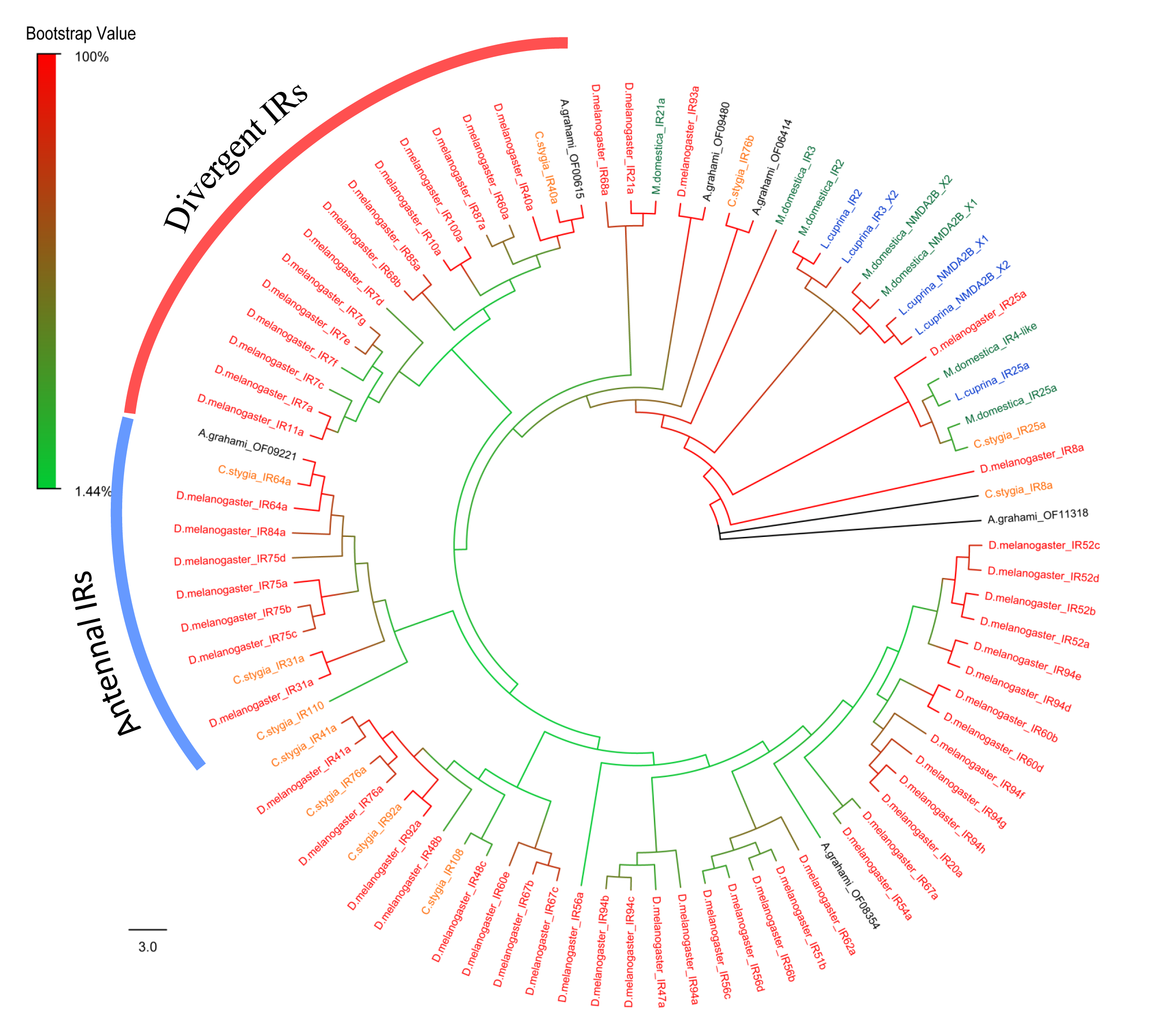

Supplement: Supplemental Information 3 — D. melanogaster: Drosophila melanogaster (red); C. stygia: Calliphora stygia (orange); M. domestica: Musca domestica (green); L. cuprina: Lucilia cuprina (blue); A. grahami: Aldrichina grahami (black). [file peerj-08-9581-s003.png]

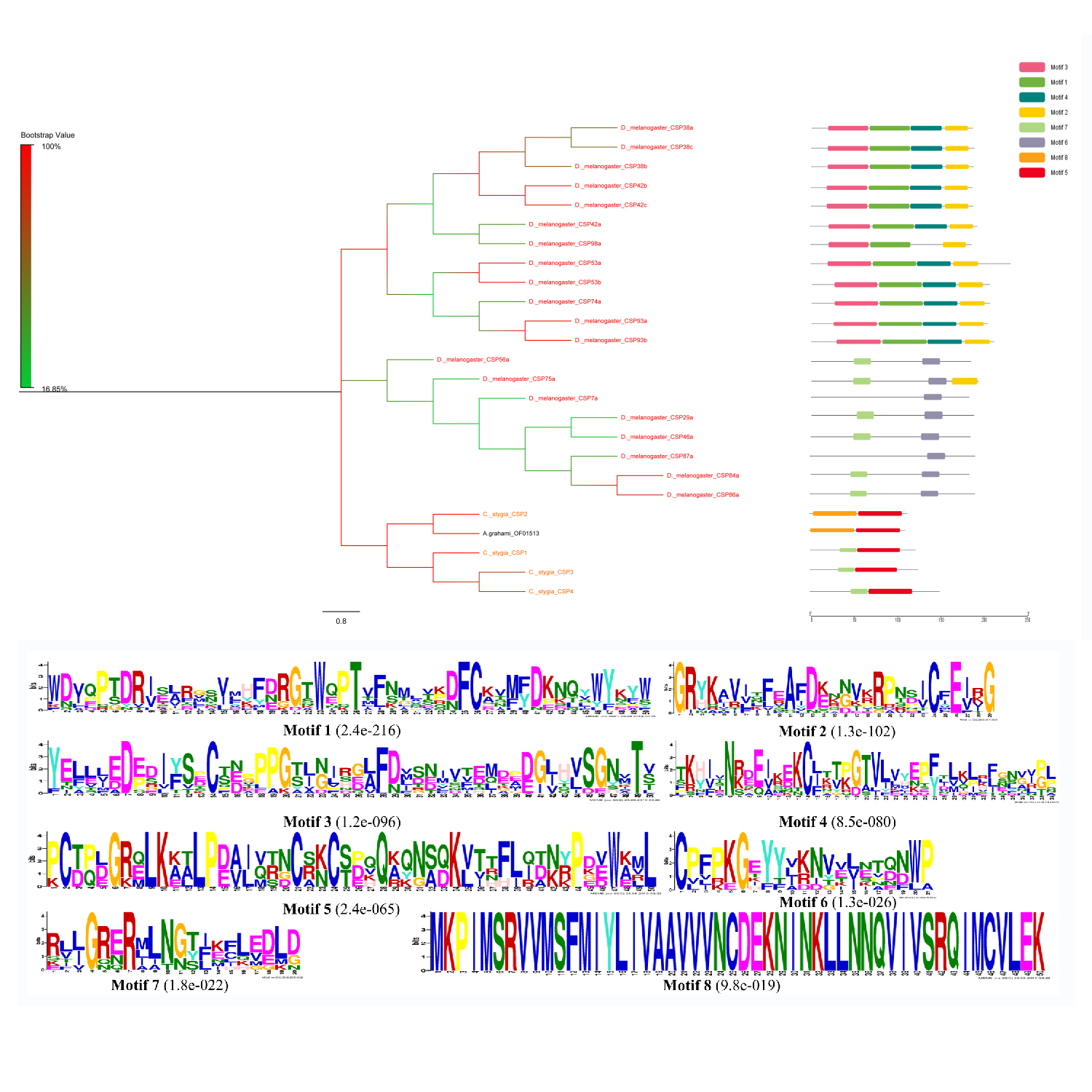

Supplement: Supplemental Information 4 — D. melanogaster: Drosophila melanogaster (red); C. stygia: Calliphora stygia (orange); M. domestica: Musca domestica (green); L. cuprina: Lucilia cuprina (blue); A. grahami: Aldrichina grahami (black). The upper parts list the eight motifs discovered in the CSPs of above species. The numbers in the boxes correspond to the numbered motifs in the upper part of the figure, where a small number indicates high conservation. [file peerj-08-9581-s004.png]

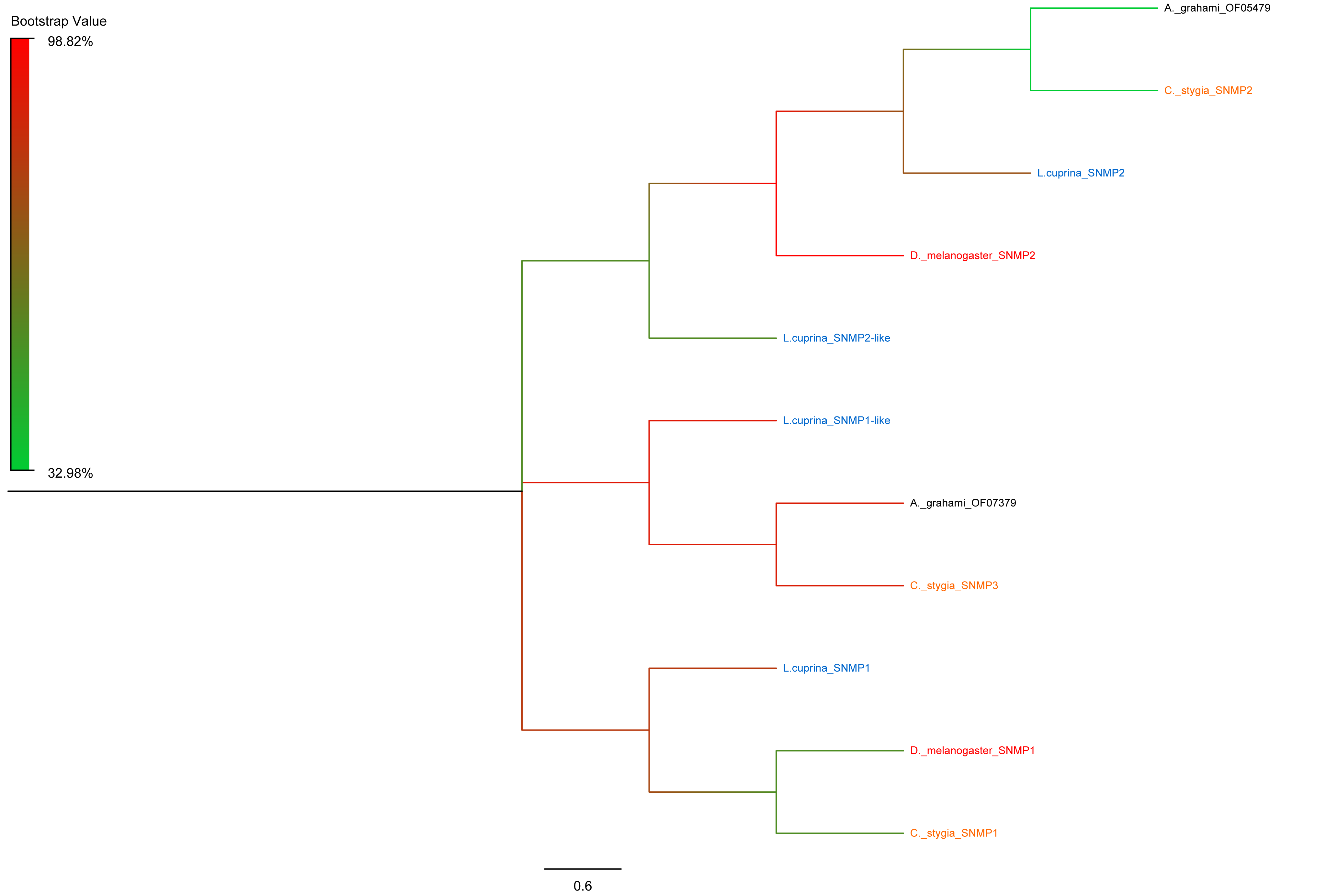

Supplement: Supplemental Information 5 — D. melanogaster: Drosophila melanogaster (red); C. stygia: Calliphora stygia (orange); L. cuprina: Lucilia cuprina (blue); A. grahami: Aldrichina grahami (black). [file peerj-08-9581-s005.png]
