# Supplementary material for: Identification of olfactory genes of a forensically important blow fly, Aldrichina grahami (Diptera: Calliphoridae)
Source: PeerJ. 2020 Aug 5;8:e9581. doi: 10.7717/peerj.9581 (PMC7414772; doi:10.7717/peerj.9581)
Supplement: Supplemental Information 6 [file peerj-08-9581-s006.docx]

Table S1: The primer of the genes of *A.grahami*

| Gene name | Primer-F(5'-3') | Primer-R(5'-3') |
| --- | --- | --- |
| OF12159 | TTAATTGCCAGCGCTGTTGC | AACATTTCATGCCGGGTGTG |
| OF08934 | AAAACCAGCATCCACAGTGG | AATGTCACTGCAGGCGTTTG |
| OF03270 | GTGTTGTGCCAGTCTCTTCG | TCCAGAACGCGGAATATTGC |
| OF03271 | TCCGGGGATTTACTACTCACC | CCTCATCAATGCCCTGGAGA |
| OF04198 | ATCTCAAGTTCCTGCCCCAA | GGTGATCGAAACTGTTGAAAGC |
| OF05347 | TGTTGTAAGTATGCCGGAAGA | AAGAATTCGGTGCCCACTTG |
| OF11636 | TGAAATAGGACTCGGGCTTGT | GAGGCGCCACTAGACAAAAG |
| OF12341 | GTGTCCAGCCTGTCGAGTAT | CCTCAGCCGAAGACAAAACC |
| OF12624 | TTTCATCGTCGAGCCTCTGT | GTGTGAGTATTGGTGCAGGC |
| OF06321 | CCGTAACACATGCCCACAAA | GGCAGCTTCACAGTGATCAT |
| OF09681 | TCGATGAGAATGCTGCCAAA | ATCACAGTCATTAGCGCCCT |
| OF00577 | TGGGCTCTTAATCCTCTGCT | GCCATTTCCAAAGATGCAGC |
| BGI_novel_G000488 | AGAAATTTGCAGCACCCGAA | CGAAAGTGCCAGCGGAAAT |
| BGI_novel_G000012 | TGCACAATAACGCTCACACT | TCACCATCTGCATCGTTGTC |
| BGI_novel_G000010 | TCCAAGGAAAAGGAGGTGGT | GACAGCAGAATTTCAGGGGC |
