# Supplementary material for: Identification of olfactory genes of a forensically important blow fly, Aldrichina grahami (Diptera: Calliphoridae)
Source: PeerJ. 2020 Aug 5;8:e9581. doi: 10.7717/peerj.9581 (PMC7414772; doi:10.7717/peerj.9581)
Supplement: Supplemental Information 7 — Sample: Sample name; Total Raw Reads (Mb): The reads amount before filtering; Total Clean Reads (Mb):The reads amount after filtering; Total Clean Bases (Gb): The total base amount after filtering; Clean Reads Q30 (%): The rate of bases which quality is greater than 30 value in clean reads; Clean Reads Ratio (%): The ratio of the amount of clean reads; Total Mapping (%): Compared with the clean reads ratio of the reference genome; Uniquely Mapping (%): Compare the clean reads ratio of the unique position of the reference genome. [file peerj-08-9581-s007.docx]

**Table 1** **Clean reads quality metrics from the *A. grahami***

| Sample | Total Raw Reads (M) | Total Clean Reads (M) | Total Clean Bases(Gb) | Clean Reads Q30(%) | Clean Reads Ratio(%) | Total Mapping(%) | Uniquely Mapping(%) |
| --- | --- | --- | --- | --- | --- | --- | --- |
| Aldrich_F_1 | 47.43 | 42.51 | 6.38 | 88.75 | 89.62 | 78.58 | 51.76 |
| Aldrich_F_2 | 48.03 | 42.9 | 6.43 | 89.12 | 89.32 | 76.49 | 51.52 |
| Aldrich_F_3 | 47.43 | 42.68 | 6.4 | 88.8 | 89.97 | 77.92 | 51.92 |
| Aldrich_M_1 | 50.95 | 45.1 | 6.76 | 89.32 | 88.52 | 75.62 | 50.13 |
| Aldrich_M_2 | 49.19 | 43.02 | 6.45 | 88.9 | 87.45 | 74.83 | 49.82 |
| Aldrich_M_3 | 47.05 | 42.3 | 6.34 | 88.91 | 89.9 | 77.88 | 51.77 |

**Sample**: Sample name; **Total Raw Reads(Mb)**: The reads amount before filtering; **Total Clean Reads(Mb)**:The reads amount after filtering; **Total Clean Bases(Gb)**: The total base amount after filtering; **Clean Reads Q30(%)**: The rate of bases which quality is greater than 30 value in clean reads; **Clean Reads Ratio(%)**: The ratio of the amount of clean reads; **Total Mapping(%)**: Compared with the clean reads ratio of the reference genome; **Uniquely Mapping(%)**: Compare the clean reads ratio of the unique position of the reference genome.
