# Supplementary material for: Identification of olfactory genes of a forensically important blow fly, Aldrichina grahami (Diptera: Calliphoridae)
Source: PeerJ. 2020 Aug 5;8:e9581. doi: 10.7717/peerj.9581 (PMC7414772; doi:10.7717/peerj.9581)
Supplement: Supplemental Information 8 — FPKM, fragments per kilobase of transcript per million mapped reads. [file peerj-08-9581-s008.docx]

Table S2: Unigenes of candidate chemosensory proteins in *A.grahami*

FPKM: Fragments per kilobase of transcript per million mapped reads
