# Supplementary material for: Identification of olfactory genes of a forensically important blow fly, Aldrichina grahami (Diptera: Calliphoridae)
Source: PeerJ. 2020 Aug 5;8:e9581. doi: 10.7717/peerj.9581 (PMC7414772; doi:10.7717/peerj.9581)
Supplement: Supplemental Information 11 [file peerj-08-9581-s011.docx]

Table S6 Enrichment Results of Differential Gene KEGG Pathway

| Gene ID | Level 1 | Level 2 | log2^(Aldrich_M/Aldrich_F)^ |
| --- | --- | --- | --- |
| BGI_novel_G000010 | Environmental Information Processing;Organismal Systems | Sensory system;Digestive system;Signal transduction | -2.093909892 |
| BGI_novel_G000012 | Environmental Information Processing;Organismal Systems | Signal transduction;Sensory system;Digestive system | -2.099637456 |
| BGI_novel_G000013 | Organismal Systems;Environmental Information Processing | Sensory system;Signal transduction;Digestive system | -1.746611994 |
| BGI_novel_G000014 | Organismal Systems;Environmental Information Processing | Sensory system;Digestive system;Signal transduction | -1.506869106 |
| BGI_novel_G000015 | Organismal Systems;Environmental Information Processing | Digestive system;Sensory system;Signal transduction | -1.586467267 |
| BGI_novel_G002169 | Cellular Processes;Organismal Systems;Environmental Information Processing | Immune system;Signal transduction;Endocrine system;Circulatory system;Environmental adaptation;Nervous system;Cellular community - eukaryotes;Digestive system;Sensory system | -1.224557927 |
| OF04198 | Environmental Information Processing;Organismal Systems;Cellular Processes;Human Diseases | Immune system;Signal transduction;Nervous system;Infectious diseases: Bacterial;Neurodegenerative diseases;Cancers: Overview;Sensory system;Substance dependence;Environmental adaptation;Endocrine system;Cancers: Specific types;Infectious diseases: Viral;Cardiovascular diseases;Circulatory system;Digestive system;Cell growth and death | 5.289692332 |
| OF10375 | Organismal Systems;Environmental Information Processing;Metabolism | Signal transduction;Immune system;Carbohydrate metabolism;Global and overview maps;Endocrine system;Sensory system | -1.42270239 |
| OF12624 | Human Diseases;Metabolism;Organismal Systems;Environmental Information Processing;Genetic Information Processing;Cellular Processes | Endocrine system;Substance dependence;Signal transduction;Sensory system;Glycan biosynthesis and metabolism;Translation;Immune system;Transport and catabolism | 3.057667313 |
